# Supplementary material for: Granulocyte Colony Stimulating Factor Expression in Breast Cancer and Its Association with Carbonic Anhydrase IX and Immune Checkpoints
Source: Cancers (Basel). 2021 Mar 1;13(5):1022. doi: 10.3390/cancers13051022 (PMC7957699; doi:10.3390/cancers13051022)
Supplement: Supplementary file 1 [file cancers-13-01022-s001.pdf]

cancers

MDPI

Article

Granulocyte colony stimulating factor expression in breast cancer and its association with carbonic anhydrase IX and immune checkpoints

Shawn C. Chafe, Nazia Riaz , Samantha Burugu, Dongxia Gao, Samuel C.Y. Leung, Anna F. Lee, Cheng-Han Lee, Shoukat Dedhar, Torsten O Nielsen

Supplementary data: cohort I:

G-CSF expression in breast cancer and its association with clinicopathological features and prognosis:

Overall, 76.7% (253/330) cases were evaluable for G-CSF expression. Of these, 46.6% tumours were high expressers. After Bonferroni corrections for multiple comparisons, no significant associations were observed between expression of G-CSF and major clinicopathological features in this cohort (Supplementary Table S2). Univariable analysis revealed that cases with G-CSF_{high} expression demonstrated a favorable trend for breast cancer specific survival (HR 0.58, CI 0.32–1.06; $p = 0.07$) and a significantly better overall survival (HR 0.51, CI 0.32–0.82; $p = 0.004$) (Supplementary Figure 1). In multivariable analysis tumours with G-CSF_{high} expression maintained the prognostic associations with primary and secondary endpoints, independent of the traditional clinicopathological features (Supplementary Table 3)

Supplementary Tables:

Table S1. Details of the antibodies and staining protocols.

Antibodies	Clone	Supplier	Antigen retrieval	Dilution	Detection	Positive controls
**Rabbit polyclonal G-CSF**	HPA001412	Sigma	HIER x 64 minutes **	1:25 × 2 h (RT)	ChromoMap DAB (Ventana)	Normal pancreas
**Mouse monoclonal CD163**	NCL-L-CD163	Novocastra	HIER **	1:500 × 2 h (RT)	ChromoMap DAB (Ventana)	Placenta
**Mouse monoclonal CAIX**	M75	Bioscience Slovakia	HIER ×30 min ***	1:50	Vector ImPRESS	Breast cancer tissue

* For all protocols, array sections at 4μm were mounted on poly-L-lysine coated glass slides and baked for an hour at 60 °C to prepare for staining on the Ventana Discovery XT automated Stainer (Ventana Medical Systems, Tucson, AZ). For negative controls, primary antibodies were replaced with PBS; ** Antigen retrieval was performed by using Cell Conditioning 1 (Ventana); HIER = heat induced epitope retrieval; *** Antigen retrieval was performed using citrate buffer.

Table S2. Cohort I: Correlation of G-CSF expression with clinicopathological features & other biomarkers.

Clinicopathological variables	G-CSF Expression		*p*-value *
	**Low (≤1)**	**High (>1)**	
**Age at diagnosis**			
< 50	40 (29.6)	51 (43.2)	0.03
≥ 50	95 (70.4)	67 (56.8)	
**Menstrual status**			0.03

Clinicopathological variables	G-CSF Expression		*p*-value *
	Low (≤1)	High (>1)	
Premenopausal	46 (34.8)	56 (48.3)	
Postmenopausal	86 (65.2)	60 (51.7)	
**Tumour size (cm)**			
≤ 2	76 (56.3)	65 (55.1)	0.85
> 2	59 (43.7)	53 (44.9)	
**Tumour grade**			
1 & 2	83 (62.4)	59 (50)	0.05
3	50 (37.6)	59 (50)	
**Axillary lymph node status**			
Negative	56 (42.7)	35 (34.7)	0.21
Positive	75 (57.3)	66 (65.3)	
**Lymphovascular invasion**			
Negative	34 (26.2)	25 (21.7)	0.42
Positive	96 (73.8)	90 (78.3)	
**ER expression**			
Negative	26 (19.4)	30 (25.4)	0.25
Positive	108 (80.6)	88 (74.6)	
**PR expression**			
< 1%	46 (34.3)	38 (32.5)	0.76
≥ 1%	88 (65.7)	79 (67.5)	
**HER2 overexpression/amplification**			
Negative	126 (94.7)	104 (92.9)	0.54
Positive	7 (5.3)	8 (7.1)	
**CK5/6 expression**			
Negative	129 (96.3)	105 (89)	0.03
Positive	5 (3.7)	13 (11)	
**EGFR expression**			
Negative	125 (92.6)	98 (83.1)	0.02
Positive	10 (7.4)	20 (16.9)	
**Ki-67 proliferation index**			
< 14%	82 (61.7)	66 (56.9)	0.45
≥ 14%	51 (38.3)	50 (43.1)	
**Breast cancer subtypes (IHC based)**			
Luminal-NOS	1 (0.8)	2 (1.9)	
Luminal A	69 (57)	60 (57.7)	0.09
Luminal B	38 (31.4)	20 (19.2)	
HER2	5 (4.1)	6 (5.8)	
Basal	8 (6.6)	16 (15.4)	
**Treatment**			
No systemic therapy	28 (20.7)	25(21.2)	
Tamoxifen only; No chemotherapy	45 (33.3)	30 (25.4)	0.21
Chemotherapy only; no hormonal therapy	21 (15.6)	30 (25.4)	
Chemotherapy + Tamoxifen	41 (30.4)	33 (28)	

* Denotes differences between low and high G-CSF groups that are significant at the Bonferroni-corrected *p*-value of <0.003 (= 0.05/14). G-CSF, granulocyte colony stimulating factor; ER, estrogen receptor; PR, progesterone receptor; EGFR, epidermal growth factor receptor; HER2, human epidermal growth factor receptor 2; CK, cytokeratin; NOS, not otherwise specified; IHC, immunohistochemistry.

Table S3. Cohort I: Multivariable analysis for breast cancer specific survival and overall survival.

Covariates	Cohort I (n = 253)			
	Breast cancer specific survival		Overall survival	
	HR (95% CI)	p-value	HR (95% CI)	p-value
**Age at diagnosis**				
< 50	1	0.15	1	0.12
≥ 50	1.65 (0.83–3.28)		2.03 (1.13–3.66)	
**Tumour size (cm)**				
≤ 2	1	0.007	1	0.10
> 2	2.72 (1.32–5.62)		1.55 (0.92–2.62)	
**Tumour grade**				
1 & 2	1	0.07	1	0.19
3	1.85 (0.96–3.56)		1.41 (0.85–2.35)	
**Axillary lymph node status**				
Negative	1	0.08	1	0.03
Positive	0.50 (0.24–1.08)		0.80 (0.45–1.43)	
**Lymphovascular invasion**				
Negative	1	0.008	1	0.45
Positive	0.39 (0.20–0.78)		0.53 (0.30–0.95)	
**G-CSF expression**				
Low (≤1)	1	0.02	1	<0.001
High (>1)	0.43 (0.21–0.87)		0.32 (0.18–0.58)	

Table S4. Cohort II: Multivariable analysis for prognostic significance of immune biomarkers within non-luminal cases with CAIX positive expression.

Covariates	BCSS	
	Non-luminal cases /CAIX+ cases	
	HR (95% CI)	p-value
**Age at diagnosis**		
< 50	1	0.22
≥ 50	0.72 (0.42–1.22)	
**Tumour size (cm)**		
≤ 2	1	0.01
> 2	2.16 (1.20–3.87)	
**Tumour grade**		
1 & 2	1	0.31
3	0.68 (0.32–1.44)	
**Axillary lymph node status**		
Negative	1	0.63
Positive	1.16 (0.63–2.13)	
**LVI**		
Negative	1	0.27
Positive	1.41 (0.77–2.58)	
**H & E stromal TILs (%)**		
< 10	1	0.001
≥ 10	0.31 (0.16–0.60)	
**Age at diagnosis**		
< 50	1	0.41
≥ 50	0.79 (0.46–1.37)	
**Tumor size (cm)**		0.02

Covariates	BCSS	
	Non-luminal cases /CAIX+ cases	
	HR (95% CI)	*p*-value
≤ 2	1	
> 2	2.01 (1.12–3.60)	
**Tumor grade**		
1 & 2	1	0.19
3	0.59 (0.27–1.29)	
**Axillary lymph node status**		
Negative	1	0.55
Positive	1.20 (0.65–2.24)	
**LVI**	1	
Negative		0.13
Positive	1.60 (0.87–2.98)	
**CD8 iTIL count**		
< 1	1	0.04
≥ 1	0.57 (0.32–0.99)	
**Age at diagnosis**		
< 50	1	0.69
≥ 50	0.88 (0.49–1.60)	
**Tumour size (cm)**	1	
≤ 2		0.13
> 2	1.60 (0.87–2.98)	
**Tumour grade**	1	
1 & 2		0.51
3	0.74 (0.31–1.79)	
**Axillary lymph node status**	1	
Negative		0.35
Positive	1.37 (0.71–2.66)	
**LVI**		
Negative	1	0.38
Positive	1.35 (0.69–2.66)	
**PD1 iTIL count**	1	
< 1		0.05
≥ 1	0.48 (0.23–1.01)	
**Age at Diagnosis**	1	
< 50		0.27
≥ 50	0.73 (0.41–1.29)	
**Tumour size (cm)**	1	
≤ 2		0.03
> 2	1.94 (1.06–3.54)	
**Tumour grade**	1	
1 & 2		0.21
3	0.59 (0.25–1.36)	
**Axillary lymph node status**		
Negative	1	0.30
Positive	1.40 (0.75–2.61)	
**LVI**		
Negative	1	0.33
Positive	1.37 (0.73–2.57)	
**FOXP3 iTIL count**	1	0.20

**Covariates**	**BCSS**	
	**Non-luminal cases /CAIX+ cases**	
	**HR (95% CI)**	***p*-value**
< 2	0.69 (0.40–1.22)	
≥ 2		
**Age at diagnosis**		
< 50	1	0.33
≥ 50	0.76 (0.43–1.32)	
**Tumour size (cm)**		
≤ 2	1	0.03
> 2	2.04 (1.09–3.80)	
**Tumour grade**		
1 & 2	1	0.37
3	0.69 (0.30–1.59)	
**Axillary lymph node status**		
Negative	1	0.24
Positive	1.46 (0.78–2.75)	
**LVI**		
Negative	1	0.69
Positive	1.14 (0.59–2.19)	
**TIM3 iTIL count**		
< 1	1	0.02
≥ 1	0.38 (0.17–0.85)	
**Age at diagnosis**		
< 50	1	0.44
≥ 50	0.79 (0.43–1.44)	
**Tumour size (cm)**		
≤ 2	1	0.02
> 2	2.11 (1.13–3.93)	
**Tumour grade**		
1 & 2	1	0.10
3	0.50 (0.22–1.55)	
**Axillary lymph node status**		
Negative	1	0.34
Positive	1.37 (0.71–2.63)	
**LVI**		
Negative	1	0.23
Positive	1.50 (0.79–2.91)	
**LAG3 iTIL count**		
< 1	1	0.02
≥ 1	0.44 (0.21–0.89)	
**Age at diagnosis**		
< 50	1	0.17
≥ 50	0.65 (0.35–1.21)	
**Tumour size (cm)**		
≤ 2	1	0.07
> 2	1.81 (0.95–3.45)	
**Tumour grade**		
1 & 2	1	0.20
3	0.58 (0.25–1.34)	
**Axillary lymph node status**		0.34

**Covariates**	**BCSS**	
	**Non-luminal cases /CAIX+ cases**	
	**HR (95% CI)**	***p*-value**
Negative	1	
Positive	1.42 (0.69–2.91)	
**LVI**		
Negative	1	0.24
Positive	1.55 (0.75–3.24)	
**PD-L1+ tumor cells (%)**		
0	1	0.02
≥ 1	0.35 (0.15–0.83)	
**Age at diagnosis**		
<50	1	0.37
≥50	0.77 (0.44–1.35)	
**Tumour size (cm)**		
≤ 2	1	0.04
> 2	1.80 (1.02–3.18)	
**Tumour grade**		
1 & 2	1	0.16
3	0.58 (0.27–1.23)	
**Axillary lymph node status**		
Negative	1	0.68
Positive	1.14 (0.62–2.08)	
**LVI**		
Negative	1	0.10
Positive	1.67 (0.91–3.08)	
**CD163+M2 macrophages**		
Sparse	1	
Moderate	0.98 (0.47–2.10)	0.97
Dense	0.83 (0.40–1.71)	0.61

Supplementary Figures:

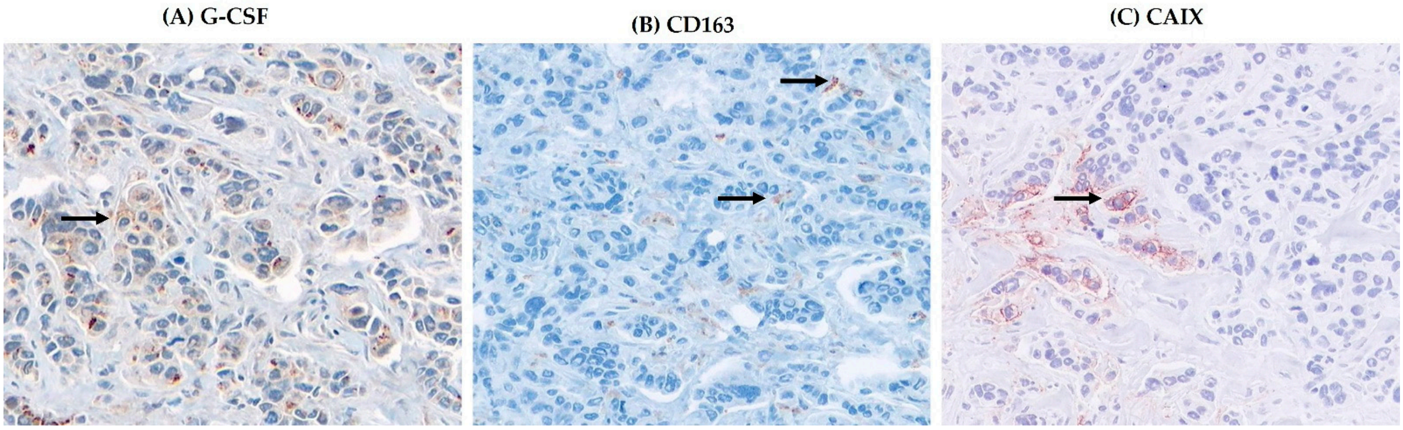

Figure S1. Representative photomicrographs for immunohistochemical staining of G-CSF, CD163 and CAIX in serial sections in core # 1715 from breast carcinoma tissue microarray (cohort II). Black arrows indicate (A) cytoplasmic expression of G-CSF (>1) on breast carcinoma cells; (B) membranous or cytoplasmic expression of CD163 on tumor associated macrophages (>5 but ≤25); and (C) membranous expression of CAIX on breast carcinoma cells (Images acquired at 200×).

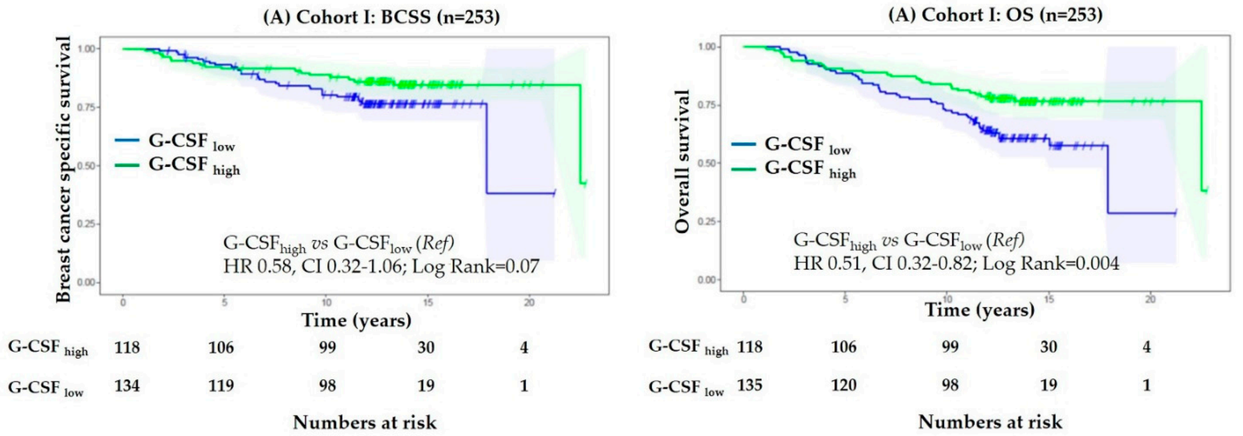

Figure S2. Cohort I: Kaplan Meier curves: Association of G-CSF expression with breast cancer specific survival (A) and overall survival (B).

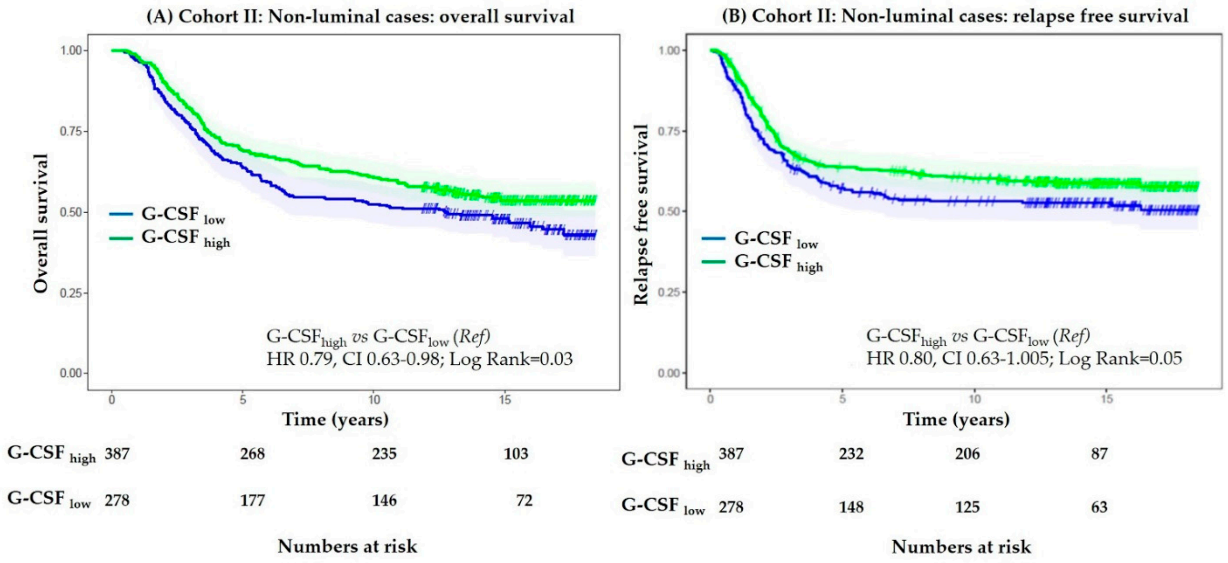

Figure S3. Cohort II: Kaplan Meier curves: Association of G-CSF expression with overall survival (A) and relapse free survival (B) in non-luminal cases**.**

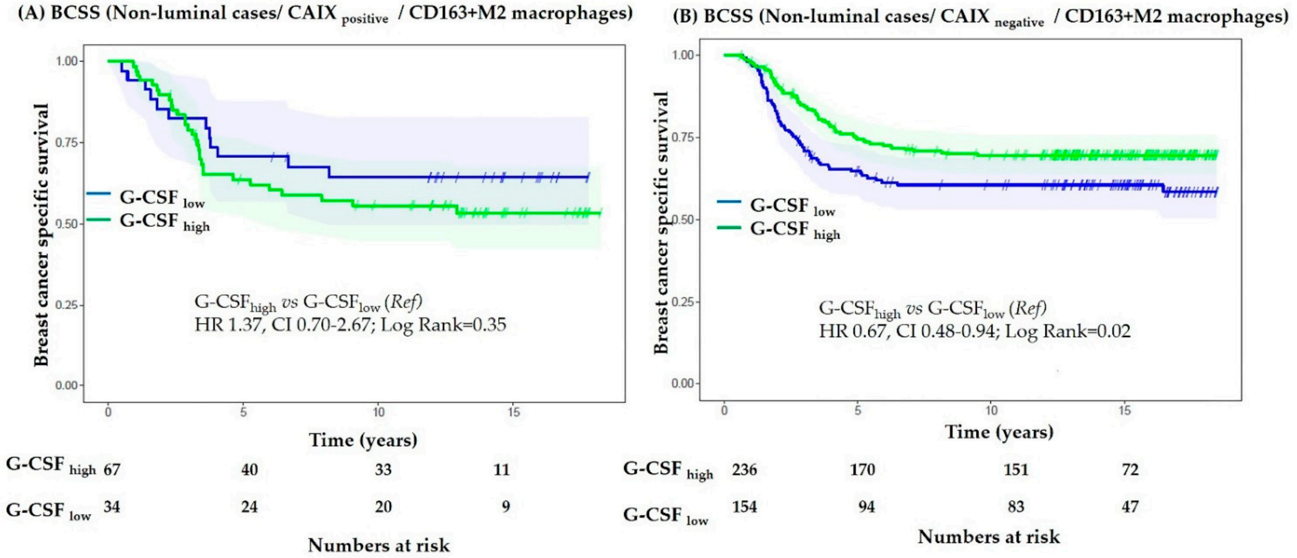

.

Figure S4. Kaplan Meier curves for association of G-CSF in CD163+ non-luminal tumors with positive (A) and negative (B) expression of CAIX.

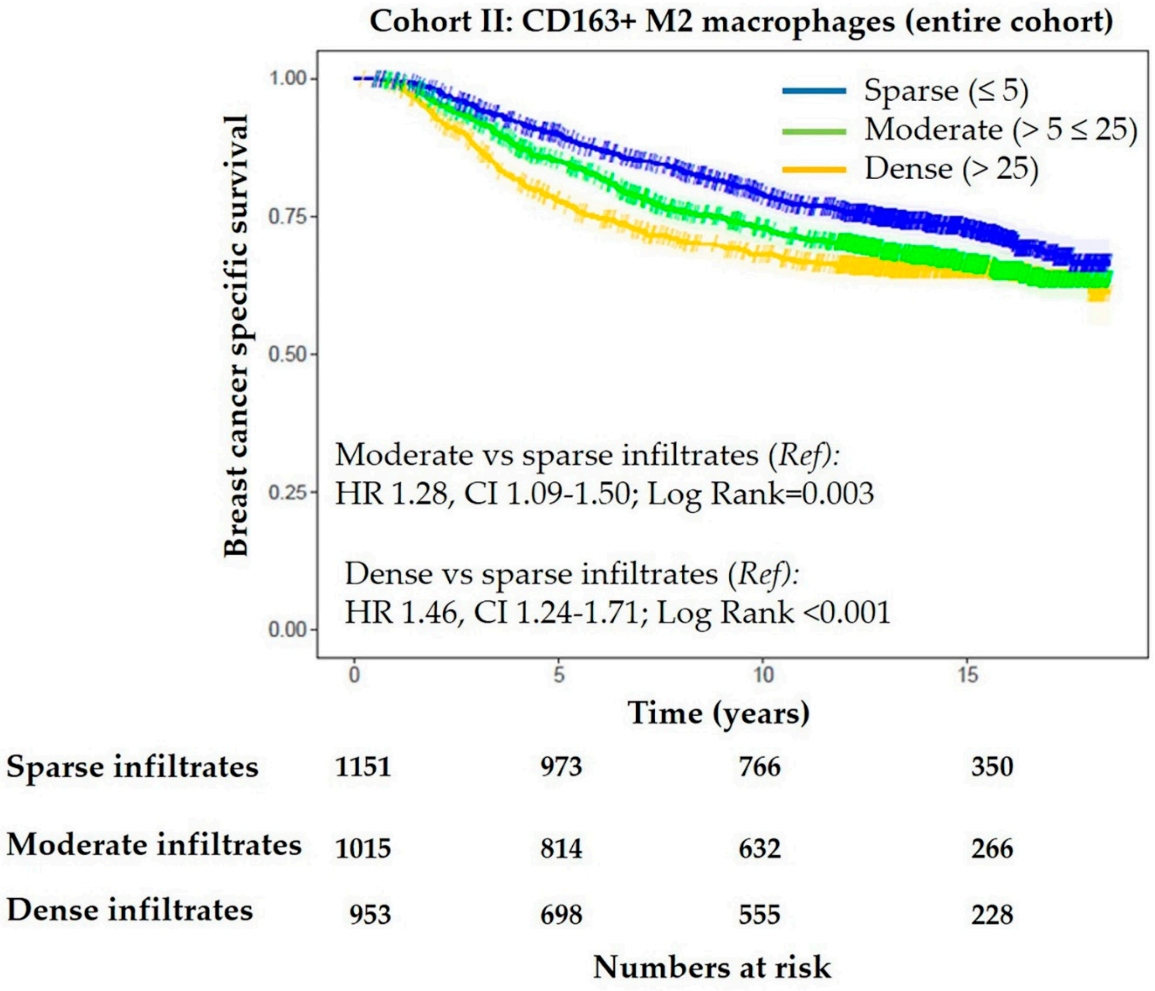

Figure S5. Kaplan Meier curves: Presence of moderate and dense infiltrates of CD163+ M2 tumor associated macrophages is associated with poor breast cancer specific survival.
